# CRISPR transcript processing: a mechanism for generating a large number of small interfering RNAs

**DOI:** 10.1186/1745-6150-7-24

**Published:** 2012-07-31

**Authors:** Marko Djordjevic, Magdalena Djordjevic, Konstantin Severinov

**Affiliations:** 1 Institute of Physiology and Biochemistry, Faculty of Biology, University of Belgrade, Belgrade, Serbia; 2 Institute of Physics Belgrade, University of Belgrade, Belgrade, Serbia; 3 Institutes of Molecular Genetics and Gene Biology, Russian Academy of Sciences, Moscow, Russia; 4 Department of Molecular Biology and Biochemistry, Rutgers, State University of New Jersey, Piscataway, USA; 5 Waksman Institute, Rutgers, State University of New Jersey, Piscataway, USA

**Keywords:** CRISPR/Cas, Transcript processing, Small RNA, CRISPR expression regulation, CRISPR/Cas response

## Abstract

**Background:**

CRISPR/Cas (Clustered Regularly Interspaced Short Palindromic Repeats/CRISPR associated sequences) is a recently discovered prokaryotic defense system against foreign DNA, including viruses and plasmids. CRISPR cassette is transcribed as a continuous transcript (pre-crRNA), which is processed by Cas proteins into small RNA molecules (crRNAs) that are responsible for defense against invading viruses. Experiments in *E. coli* report that overexpression of *cas* genes generates a large number of crRNAs, from only few pre-crRNAs.

**Results:**

We here develop a minimal model of CRISPR processing, which we parameterize based on available experimental data. From the model, we show that the system can generate a large amount of crRNAs, based on only a small decrease in the amount of pre-crRNAs. The relationship between the decrease of pre-crRNAs and the increase of crRNAs corresponds to strong linear amplification. Interestingly, this strong amplification crucially depends on fast non-specific degradation of pre-crRNA by an unidentified nuclease. We show that overexpression of *cas* genes above a certain level does not result in further increase of crRNA, but that this saturation can be relieved if the rate of CRISPR transcription is increased. We furthermore show that a small increase of CRISPR transcription rate can substantially decrease the extent of *cas* gene activation necessary to achieve a desired amount of crRNA.

**Conclusions:**

The simple mathematical model developed here is able to explain existing experimental observations on CRISPR transcript processing in *Escherichia coli*. The model shows that a competition between specific pre-crRNA processing and non-specific degradation determines the steady-state levels of crRNA and is responsible for strong linear amplification of crRNAs when *cas* genes are overexpressed. The model further shows how disappearance of only a few pre-crRNA molecules normally present in the cell can lead to a large (two orders of magnitude) increase of crRNAs upon *cas* overexpression. A crucial ingredient of this large increase is fast non-specific degradation by an unspecified nuclease, which suggests that a yet unidentified nuclease(s) is a major control element of CRISPR response. Transcriptional regulation may be another important control mechanism, as it can either increase the amount of generated pre-crRNA, or alter the level of *cas* gene activity.

**Reviewers:**

This article was reviewed by Mikhail Gelfand, Eugene Koonin and L Aravind.

## Background

CRISPR (Clustered Regularly Interspaced Short Palindromic Repeats) cassettes are present in almost every known archaeal genome and in about half of the known bacterial genomes [1-3]. A CRISPR cassette consists of identical direct repeats of about 30 bp in length, interspaced with spacers of similar length [4]. The length of different spacers within the same cassette is the same, while sequences of these spacers are different. In many organisms, these spacer sequences closely match sequences of bacteriophages (bacterial viruses) infecting this or closely related organisms [5-7]. It was recently discovered that CRISPR/Cas loci function as an adaptive immunity system, which is responsible for defending prokaryotic cell against viruses and plasmids [8,9]. A match between a CRISPR spacer and sequence in invading DNA provides immunity to infection [5-9].

In *E. coli*, promoters that transcribe CRISPR cassettes and *cas* genes are distinct, and are (at least under normal growth conditions) considered to be poorly active due to repression by H-NS transcription factor [10]. The entire CRISPR cassette is transcribed as a long continuous transcript [10,11], which is then processed by one of the Cas proteins (CasE), to small RNA molecules (crRNAs) [11,12]. Once crRNAs are generated, they bind a large multisubunit complex of Cas proteins called Cascade and target it to matching DNA of viruses and plasmids, ultimately leading to its destruction [13].

While it is clear that CRISPR/Cas system in *E. coli* is functional [11,14], virus infection in itself appears not to lead to system induction (at least under normal conditions) [15], and physiological conditions under which the system is induced yet have to be determined [13]. Consequently, functioning of this system has been investigated by either artificial overexpression of *cas* genes and CRISPR array from plasmids, or by inhibition of H-NS repression of *cas* and CRISPR promoters [11,12,16]. In a recent study, *cas* genes were overexpressed in *E. coli*, and resulting changes in the levels of pre-crRNAs and crRNAs were quantitatively measured [11]. In cells with endogenous (uninduced) *cas* expression, the abundance of pre-crRNA and individual crRNAs was low, below 10 molecules per cell. When CasE was overexpressed, the abundance of crRNAs increased dramatically, to about 1000 molecules per cell, while pre-crRNA became undetectable. There is, therefore, a large (at least two orders of magnitude) increase in abundance of individual crRNAs, accompanied by a much smaller (less than tenfold) decrease of pre-crRNA. It remains unclear if (and by what model) this strong amplification of crRNA upon *cas* overexpression can be explained. Answering this question is a major goal of this paper.

Furthermore, the experiments discussed above correspond to measurements where *cas* genes and CRISPR arrays are overexpressed to a fixed level [10-12,16]. On the other hand, it is important to explore how changes of the relevant parameters affect generation of crRNAs, since such understanding can provide important clues about the mechanism of the endogenous system induction. Finally, the available experiments correspond to steady-state measurements of transcript amounts, i.e. come from measurements taken long after *cas* genes overexpression has been induced. However, the steady-state regime may not be directly relevant for system function under natural conditions, where the amount of generated crRNA immediately after system induction (i.e., for example, after virus infection) may be more relevant. While it is hard to experimentally assess either different levels of parameter changes or kinetics of the transcript accumulation, this analysis can be readily done through mathematical modeling, which is another major goal of this paper.

We will in this paper present a simple mathematical model of CRISPR expression that is able to *i)* determine biochemical parameters relevant for CRISPR transcript processing, *ii)* explain the observed large amplification of crRNAs, *iii)* assess how different levels of change in the transcription and processing rates affect steady-state levels and kinetics of crRNA accumulation.

## Results

### Model definition

In this section, we will propose a simple model of CRISPR transcript processing. The model is in accordance with the following experimental observations:

i) Endogenous (uninduced) levels of pre-crRNAs and crRNAs are low (~10 copies per cell) [11,12,16], which was reported to be a consequence of repression of *cas* and (to a smaller extent) CRISPR promoters by H-NS [10].

ii) One of the Cas proteins (CasE) is responsible for processing pre-crRNAs to crRNAs [11,12]. When CasE is overexpressed, the amount of crRNAs increases for about two orders of magnitude, while the amount of pre-crRNAs drops to only few transcripts per cell [11]. Overexpression of CasE affects only the processing rate of pre-crRNA to crRNA, since it has been shown [11] that CasE does not influence either pre-crRNA transcription rate or crRNA stability.

iii) In addition to being processed by CasE, pre-crRNA is also degraded by an unspecified nuclease [10,11]. As a consequence of this degradation, pre-crRNA decays with a half-life of ~1 min without generating crRNAs. On the other hand, crRNAs are observed to be much more stable [11].

iv) It is currently unclear how CRISPR/Cas system is induced under natural conditions [13]. It was, however, showed that the repression of the *cas* promoter by H-NS can be relieved by a transcription activator (LeuO) [16]. It was consequently proposed that the endogenous system induction may involve activation of *cas* and (to a smaller extent) CRISPR promoters, through abolishment of H-NS repression [10].

**Figure 1:**
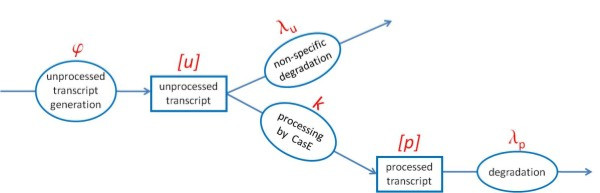
**Model of CRISPR transcript processing.** The unprocessed transcripts (pre-crRNAs) are generated with rate *ϕ*, and are consequently either (non-specifically) degraded with rate λ_*u*_, or processed to crRNAs by CasE with rate *k*. crRNAs are then degraded with rate λ_*p*_. Concentrations of pre-crRNAs (unprocessed transcripts) and crRNAs (processed transcripts) are denoted as, respectively, [*u*] and [*p*].

The simplest model of CRISPR transcript processing, which is in accordance with the experimental observations summarized above, is schematically shown in Figure 1. In the scheme, we denote concentrations of the unprocessed (pre-crRNA) and processed (crRNA) transcripts as, respectively, *u* and *p*. The unprocessed transcripts (pre-crRNAs) are transcribed with rate *ϕ*; pre-crRNAs are further either non-specifically degraded with rate *λ*_u_, or processed by CasE with rate *k*. By non-specific degradation, we mean degradation that does not lead to accumulation of crRNA. Processing of pre-crRNA by CasE leads to formation of individual crRNAs, which are further degraded with rate *λ*_p_. Based on the experimental results [11], we take *λ*_u_ ~ 1 min^-1^, *λ*_p_ ~ 1/100 min^-1^, and *u* ~ *p* ~ 10.

While the uninduced values of pre-crRNA transcription and processing rates (*ϕ* and *k*) have not been experimentally measured, they can be determined from equations that describe kinetics of the system in Figure 1 (see the next section). When the system is induced, both *k* and *ϕ* can be increased. Since CasE is solely responsible for processing of pre-crRNA to crRNA, the value of the processing rate *k* depends on the amount of CasE. Consequently, the increase of *k* is due to increased amount of CasE, which is a consequence of a larger transcription activity of *cas* promoters. Similarly, *ϕ* can be increased if the CRISPR promoter becomes more active.

In the next subsection, we will show that the simple model, schematically shown in Figure 1, together with experimentally inferred parameter values summarized above, can indeed explain the observed large crRNA amplification upon induction of *cas* gene expression. We will afterwards explore kinetics of crRNA generation, and investigate how modulation of pre-crRNA transcription and processing rate (*ϕ* and *k*) affects generated crRNA amounts.

### Uninduced system parameters

Starting from equations that describe the system kinetics (see Methods), it is straightforward to obtain expressions for uninduced values of pre-crRNA transcription and processing rates (*ϕ* and *k*):

(0.1) $$\phi =\lambda_{u}[u]+\lambda_{p}[p]\text{,}$$

(0.2) $$k=\lambda_{p}\frac{\left[p\right]}{\left[u\right]}$$

In the equations above [*u*] and [*p*] are, respectively, (uninduced) steady state amounts of pre-crRNA and crRNA, while *λ*_u_ and *λ*_p_ are defined in Figure 1.

By using the numerical values stated in the previous section, from Eq. (0.1) we obtain *ϕ*~10*λ*_u_~10 min^-1^. This value corresponds to a moderately strong transcription activity; note that transcription activity of very strong rRNA promoters is ~60 min^-1^, while basal activity of a very weak uninduced λ_PRM_ promoter is ~1/7 min^-1^[17]. It is interesting that in experimental studies the CRISPR promoter was labeled as weak, based on measured small amount of pre-crRNA [10,11]. The small amount of pre-crRNA is actually a consequence of a high non-specific decay rate of pre-crRNA (note that pre-crRNA half life is ~1 min), which has to be matched by the relatively high activity of the CRISPR promoter. The moderately high transcription rate of the CRISPR promoter implies a weak repression of this promoter by H-NS, which is consistent with the experimental finding that repression of the CRISPR promoter by H-NS is significantly weaker compared to the repression of the *cas* promoter [10,16].

Similarly, by using numerical values from the previous subsection and Eq. (0.2), we obtain *k*~*λ*_p_ ~ 1/100 min^-1^. Therefore, pre-crRNA to crRNA processing rate (*k*) is an order of magnitude smaller than pre-crRNA decay rate (*λ*_u_). Due to this, when the system is uninduced, almost all generated pre-crRNA is rapidly degraded (see Figure 1), which results in small crRNA amounts, despite the moderately high transcription rate (*ϕ*) of the uninduced promoter. As we will show in the next subsection, when the system is induced and *k* is increased, the system switches from the state in which almost all of the generated pre-crRNA is degraded, to the state in which most of the generated pre-crRNA is processed to crRNA.

### Overexpression of *cas* genes

We next analyze the experiments in which CasE is overexpressed, and the transcript numbers are quantified [11]. In these experiments, the number of pre-crRNA and crRNA transcripts has been measured both before and after the system induction. In the analysis below, we assume that overexpression of CasE leads to an increase of pre-crRNA to crRNA processing rate from *k* to *k'*, while it has been experimentally shown that the rest of the parameters remain unchanged (see above). Furthermore, we denote pre-crRNA and crRNA amounts upon CasE overexpression as, respectively, *u*' and *p*'. Note that primes in our notation correspond to the quantity values after the system induction, rather than to derivatives.

We aim to understand the large amplification of crRNA, where, upon CasE overexpression, a decrease from about ~10 pre-crRNA transcripts present in uninduced cells leads to about two orders of magnitude increase in the amount of crRNA (~1000 transcripts). To that end, it is useful to derive a relationship between the changes in the number of crRNAs (Δ[*p*] ≡ [*p*]'-[*p*]) and pre-crRNAs (Δ[*u*] ≡ [*u*]'-[*u*]). By using the equations for the system kinetics (see Methods), one can derive the following (exact) relation:

(0.3) $$\Delta [p]=-\frac{\lambda_{u}}{\lambda_{p}}\Delta [u]$$

Note that the minus sign indicates that the decrease in the number of unprocessed transcripts (pre-crRNA), leads to an increase in the number of processed transcripts (crRNA). From the relationship above follows that the crRNA increase is directly proportional to the pre-crRNA decrease, where the constant of proportionality is equal to 100 (λ_u_/λ_p_ ~ 100 - see the previous section). This large constant of proportionality in Eq. (0.3) explains the experimentally observed large amplification of crRNA upon CasE overexpression. That is, according to Eq. (0.3), ~10 molecule decrease in pre-crRNA (Δ[*u*] ~ 10), leads to two orders of magnitude larger increase in crRNA (Δ[*p*] ~ 1000), as observed in the experiments. Therefore, Eq. (0.3) shows that the system acts as a strong linear amplifier, where the increase of crRNA is directly proportional to the decrease of pre-crRNA, and where a small number of pre-crRNAs are amplified to a large number of crRNAs.

Experiments also report that, upon Cas overexpression, the amount of pre-crRNA decreases for about one order of magnitude, which allows estimating the extent of increase of pre-crRNA to crRNA processing rate (*k*). From equations that describe the system kinetics (see Methods), it is straightforward to show that the relative decrease of pre-crRNA amount is given by

(0.4) $$\frac{\left[u\right]}{[u]'}=\frac{\lambda_{u}+k'}{\lambda_{u}+k}\text{.}$$

It is experimentally observed that [*u*]/[*u*]*'* ~ 10, so from Eq. (0.4) follows *k'* ~ 10(*λ*_*u*_ *+ k*). Since we obtained that $k\ll \lambda_{u}$, it follows that *k' ~ 10λ*_*u*_, i.e. due to the overexpression of CasE, the processing rate becomes for an order of magnitude larger than pre-crRNA decay rate. Therefore, the overexpression of CasE makes the system switch from the state in which almost all of the generated pre-crRNA is degraded, to the state where most of the generated pre-crRNA is processed to crRNA.

We will below use the values of the system parameters that were estimated above, in order to numerically investigate kinetics of the transcript accumulation. To investigate the kinetics, we will simulate the system both deterministically and stochastically; we perform the stochastic simulations since the number of uninduced pre-crRNA and crRNA molecules are small, and since the number of pre-crRNA molecules becomes even smaller as CasE is overexpressed. However, we will see in the subsequent figures that the stochastic and deterministic results are in agreement with each other, which validates that the simple analytic expressions that we derive (e.g. Eq. (0.3)) can be used to describe the system.

**Figure 2:**
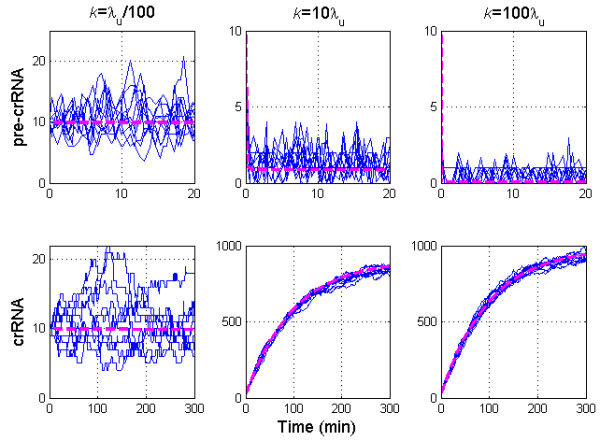
**Increase of pre-crRNA processing rate.** The first and the second row in the panel correspond, respectively, to the number of pre-crRNA and crRNA molecules. The first, the second, and the third column correspond, respectively, to A), B) and C). The deterministic simulation corresponds to the magenta dashed line, while ten simulated stochastic trajectories correspond to the full blue curves. The parameter values are as experimentally measured, or as inferred from the measurements by the analysis: $\lambda_{u}=1\;\min^{-1}$, $\lambda_{p}=1/100\min^{-1}$, $k=1/100\min^{-1}$, $\phi =10\min^{-1}$, $\left[u\right]=\left[p\right]=10$. The system is induced so that *ϕ* remains constant, while pre-crRNA processing rate (*k*): **A)** remains the same as the uninduced value, **B)** increases for three orders of magnitude (as in CasE overexpression experiments in [11]), **C)** increases for an additional order of magnitude relative to B). The figure shows that CasE overexpression can lead to a large generation of crRNA, but that increase of CasE above some value does not lead to an additional increase of crRNA amount (the saturation of crRNA).

We first numerically investigate how the amount of unprocessed and processed transcripts change as *k* is increased (i.e. as CasE is overexpressed). Stochastic simulations are performed by using Gillespie stochastic simulation algorithm [18], and stochastic trajectories are shown together with the deterministic curves. Figure 2A corresponds to the uninduced system, where the uninduced system parameters (see the previous section) lead to the experimentally observed steady state values (*u* *~* *p* *~* 10). In Figure 2B, we increase the value of *k* 1000 times; note that this *k* increase corresponds to CasE overexpression in [11] (see above). We see that for such *k* increase *u* drops to a very small amount (few transcripts per cell), while *p* increases for about two orders of magnitude, consistently with the experimental observations. In Figure 2C, *k* is increased for an additional order of magnitude (i.e. 10000 fold relative to the uninduced value). This additional increase of *k* leads to even smaller amount of pre-crRNA, while the amount of crRNA increases for an additional small value (see the discussion below).

The results in Figures 2B and 2C clearly support Eq. (0.3). That is, in both of the panels, the steady-state amount of pre-crRNA decreases to very small levels (Δu∼-10), which leads to about two orders of magnitude increase of steady-state crRNA amount (Δp∼1000). Note that this is in accordance with Eq. (0.3), given that the constant of proportionality between *Δu* and *Δp* equals 100 ($\lambda_{u}/\lambda_{p}=100$). Furthermore, both the decrease of *Δu*, and the increase of *Δp*, are somewhat larger in Figure 2C compared to Figure 2B, which is again consistent with the direct proportionality in Eq. (0.3). Therefore, both analytical and numerical results show that small pre-crRNA decrease leads to a large crRNA increase upon CasE over-overexpression. Interestingly, this strong amplification crucially depends on loss of pre-crRNA through fast non-specific degradation, i.e. on large $\lambda_{u}/\lambda_{p}$ratio (see Eq. (0.3)).

**Figure 3:**
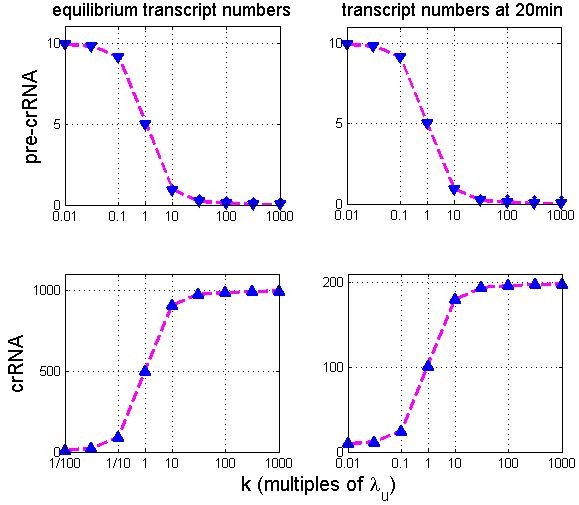
**Kinetics of crRNA accumulation.** The figure shows how pre-crRNA (the first row) and crRNA (the second row) amounts change as pre-crRNA processing rate (*k*) is increased. CRISPR transcription rate remains constant and has the same value as in Figure 2. The first and the second column correspond, respectively, to **A)** equilibrium transcript amounts and **B)** transcript amounts at 20 min post-induction. The horizontal axes in the figure correspond to *k* in multiples of *λ*_u_, where *k* changes from the uninduced value (*λ*_u_/100) to a very high value (1000*λ*_u_). The points on the horizontal axes are, for clearer presentation, plotted equidistantly, and correspond to *k* (in multiples of *λ*_u_) values of: (1/100, 1/50, 1/10, 1, 10, 50, 100, 500, 1000). The magenta line and the blue triangles correspond, respectively, to the stochastic and the deterministic simulations. The figure confirms the saturation effects observed in Figure 3, and suggests that the system is able to generate substantial crRNA amounts soon after its induction.

Furthermore, we note that the increase of *k* for one order of magnitude between Figures 2B and 2C, leads to only small additional increase of crRNA (relative to the one in Figure 2B), which we further refer to as saturation of crRNA upon increase of pre-crRNA processing rate. To additionally investigate this saturation, in Figure 3A we systematically predict the effect of *k* increase on unprocessed (pre-crRNA) and processed (crRNA) transcript amounts. We see that, as *k* is increased beyond 1000 fold, the amounts of both pre-crRNA and crRNA reach saturation; i.e. pre-crRNA and crRNA amounts do not significantly change with further increase of k. The saturation value of crRNA increase corresponds to ~100 fold.

To analytically understand the observed saturation of crRNA upon *k* increase, it is straightforward to derive (see Methods) the relative increase of crRNA, as pre-crRNA processing rate is increased from *k* to *k'*:

(0.5) Δpp=λs/k+1λs/k'+1−1

From the above equation, it follows that as *k'* becomes significantly larger than $\lambda_{s}$ (i.e. $k'≳10\lambda_{s}$), Δ[*p*]/[*p*] no longer depends on *k'*. Δp/p then reaches saturation, i.e. approaches *λ*_*s*_/*k*. Since *λ*_*s*_ ~ 100 *k*, the saturation is reached when pre-crRNA processing rate is increased for more than 1000 times, as a result of which Δ[*p*]/[*p*] increases for about two orders of magnitude.

Finally, in Figure 3B, we investigate in more detail kinetics of crRNA accumulation. Figure 2 shows that the steady state is reached relatively slowly, i.e. ~300 min after the system induction. However, when a virulent phage infects *E. coli*, the cell lysis is typically complete much before 300 min post-infection; e.g. for the well known *E. coli* T7 and T3 phages, the cell lysis starts at ~20 min post-infection, with complete shot-off of host functions occurring much earlier [19]. Therefore, the steady state crRNA levels are likely not directly relevant for *E. coli* defense against phage infection. Due to this, in Figure 3B, we estimate crRNA levels at 20 min after the system induction. We see that, similarly to Figure 3A, as *k* is increased more than 1000 fold, crRNA amount at 20 min reaches saturation. While these saturation levels (~200 transcripts) are significantly smaller compared to the steady state values, they are still much larger than crRNA levels at which a partial protection against phage infection is observed (~10 crRNA transcripts as per [11]). Therefore, activation of *cas* expression leads to a rapid accumulation of crRNA, which suggests such activation can lead to an effective protection against phage infection.

### Joint overexpression of CRISPR and *cas* genes

We next consider what happens if transcription of both *cas* genes and CRISPR array is activated. This analysis is, in part, motivated by reported repression of *cas* and (to a smaller extent) CRISPR promoters by H-NS, and by a model which proposes that the system is induced by abolishing this repression [10]. Activation of *cas* genes and CRISPR array transcription leads to increasing of both pre-crRNA processing rate (we assume that *k* increases to *k'*) and CRISPR transcription rate (we assume that *ϕ* increases to *ϕ*'). It is straightforward to derive (see Methods) that upon increase of both *ϕ* and *k*, the amount of generated crRNA is given by:

(0.6) $$\frac{\Delta \left[p\right]}{\left[p\right]}=\frac{\left(\lambda_{s}/k+1\right)}{\left(\lambda_{s}/k'+1\right)}\frac{\phi '}{\phi }-1$$

From Eq. (0.6), we see that relative increase in crRNA depends linearly on relative increase of CRISPR transcription rate (*ϕ*'/*ϕ*). From this follows that the saturation in crRNA due to increase of only *k*, which was discussed in the previous subsection, can be relieved if *ϕ* is increased as well.

**Figure 4:**
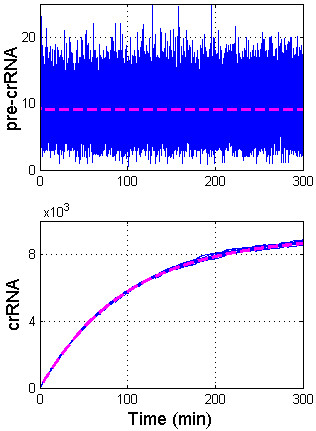
**Joint increase of k and *ϕ*.** The figure shows how pre-crRNA (the first row) and crRNA (the second row) change as *k* is increased for three orders of magnitude (the saturation value - see Figure 2), while *ϕ* is increased for one order of magnitude. The initial conditions and pre-crRNA and crRNA decay rates (*λ*_u_ and *λ*_p_) are the same as in Figure 2. The figure shows that saturation in crRNA amounts (due to increase of only *k*) can be relieved if *ϕ* is increased as well, which leads to a very large amount of generated crRNA.

Increase of crRNA due to joint increase of *k* and *ϕ* is numerically investigated in Figure 4. In this figure, *k* is increased for the amount that corresponds to the saturation (see the previous subsection), while *ϕ* is increased tenfold. Note that the tenfold increase in *ϕ* approaches maximal biochemically realistic value, since the basal *ϕ* value is already moderately high (~10 min^-1^), while the transcription rate of very strong rRNA promoters is for about one order of magnitude higher [17]. We see that such induction strategy leads to an even higher increase in the amount of generated steady-state crRNA (~10^3^ fold relative increase of crRNA upon induction); similarly, the amount of generated crRNA soon after the induction (e.g. at 20 min post-induction) - which may be relevant for defense against bacteriophages - is much higher than the minimal crRNA amount (~10 transcripts) necessary for partial protection against viruses [11].

**Figure 5:**
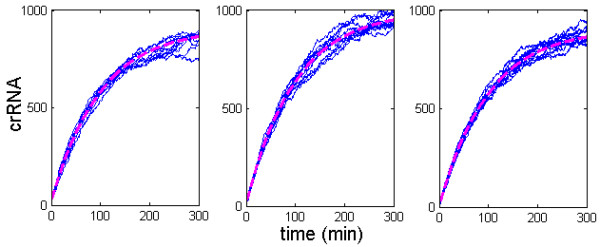
**Reducing the increase of *k *through increase of ϕ.** The figure shows increase of crRNA as **A)** pre-crRNA processing rate (*k*) is increased 1000 fold relative to the uninduced value, while CRISPR transcription rate (*ϕ*) is kept unchanged, **B)** k is increased 100 fold, while *ϕ* is increased twofold, **C)** both *k* and *ϕ* are increased 10 fold. The figure shows that a moderate increase in *ϕ* allows to substantially reduce the increase in *k*, while still achieving the same increase in crRNA amount.

In Figure 4, CRISPR transcription was increased in order to relieve the saturation due to increase of only *k* (compare with Figure 2C), and *ϕ* was increased for a maximal biochemically realistic value. Consequently, crRNA amount in Figure 4 roughly corresponds to the maximal value that can be generated by the system. On the other hand, an increase in CRISPR transcription can be also used to substantially reduce the increase in pre-crRNA processing rate, while still achieving the same increase in generated crRNAs. This possibility is explored in Figure 5, which is, in part, motivated by experiments in which H-NS repression of *cas* and CRISPR promoters is abolished [10,16]. Upon this abolishment, the amount of crRNA is increased for about two orders of magnitude, i.e. for the similar value as in CasE overexpression experiments [11,16].

Figure 5 demonstrates that the two orders of magnitude increase of crRNA can be achieved through very different levels of increase of pre-crRNA processing rate *k*, if CRISPR transcription rate is allowed to increase as well. Accordingly, the three panels in Figure 5, show roughly the same (two orders of magnitude) increase in crRNA levels, which are achieved in the following way; *i*) in Figure 5A, *k* is increased for three orders of magnitude, without increase of *ϕ*, *ii*) in Figure 5B, *k* is increased for two orders of magnitude, while *ϕ* is increased two times, *iii*) in Figure 5C, both *k* and *ϕ* are increased for one order of magnitude.

Figure 5 demonstrates that large amounts of crRNA can be generated without a large CasE overexpression - which is characteristic for the (artificial) overexpression experiments - as long as CRISPR array transcription is increased for a much smaller amount. While conditions of natural CRISPR/Cas induction are currently unclear [13], it is likely that activation of the CRISPR array promoter is much weaker compared to the activation of the *cas* promoter (note that repression of the *cas* promoter by H-NS was found to be significantly stronger than repression of the CRISPR promoter) [10,16]. This, therefore, suggests that conditions of natural system induction might roughly correspond to Figure 5B (the increase of *k* that is much larger than the increase of *ϕ*).

## Discussion

We here proposed a simple model of CRISPR transcript processing. We used this model, together with previous experimental measurements, to infer all the parameters that characterize the uninduced system. We showed that our model can explain the experimental observation that CasE-dependent decrease of very low initial steady-state level of *E. coli* pre-crRNA leads to a very large increase of crRNA abundance. Interestingly, this observation is a direct consequence of fast non-specific (i.e., not leading to crRNA) degradation of pre-crRNA. Our results, therefore, strongly suggest that non-specific degradation by an yet unidentified nuclease is a major control element of CRISPR expression and CRISPR/Cas response.

It is interesting to note that while effects of activation of *cas* gene transcription on CRISPR/Cas system were extensively studied, there is a lack of such studies for activation of CRISPR array transcription. Specifically, changes of pre-crRNA and crRNA amounts were quantitated only for *cas* gene overexpression, but not for CRISPR array overexpression [11]. Furthermore, while effects of *cas* gene overexpression on host protection against phage infection were measured [11], there is no such analysis for CRISPR array overexpression. That is, while in [12] it was shown that joint overexpression of *cas* genes and CRISPR array leads to efficient protection against bacteriophage infection, it is unclear what additional protection is provided due to CRISPR array overepression. Finally, activation by LeuO (a transcription regulator that abolishes H-NS repression) was studied for the *cas* promoter [16], but remains to be investigated for the CRISPR promoter.

Contrary to the almost complete emphasis on activation of *cas* gene transcription, the results presented here indicate that activation of CRISPR array transcription may be an important mechanism of CRISPR/Cas response. That is, we showed that there is a saturation of generated crRNA upon overexpression of only *cas* genes, i.e. that the amount of crRNA stops to increase when the rate of pre-crRNA processing is increased above certain level. This saturation is relieved when the rate of CRISPR transcription is increased as well, and we showed that a joint increase in transcription rates of *cas* and CRISPR promoters can lead to a very large (three orders of magnitude) increase of steady state crRNA levels. We, moreover, obtained that a substantial amount of crRNAs can be generated soon after the system induction, which suggests that the system may be capable for efficient protection against viruses under natural conditions. Unlike the situation observed in other bacteria, *E. coli* CRISPR spacers for the most part do no match sequences in known phages or plasmids. Yet, numerous data show that *E. coli* CRISPR/Cas system is functional once appropriate spacers are introduced by means of genetic engineering [12,20]. Presumably, the mechanism of CRISPR transcript processing, which was analyzed here, is relevant for protection against *E. coli* phages that are yet to be identified [21].

As a further support of potential importance of CRISPR array regulation, we showed that a modest increase of CRISPR transcription rate can substantially decrease for how much pre-crRNA processing rate needs to increase in order to achieve a desired crRNA amount. For example, as small as twofold increase of CRISPR transcription rate allows reducing for one order of magnitude the pre-crRNA processing rate needed to achieve the two orders of magnitude increase of crRNAs (the increase observed when H-NS repression is abolished). Since repression of *cas* promoters by H-NS was found to be significantly stronger than the repression of CRISPR promoters, the regime in which the increase of pre-crRNA processing rate is significantly larger compared to the increase of CRISPR transcription rate may be directly relevant for natural system induction.

## Conclusions

We here developed a simple model of CRISPR transcript processing, and showed that this model is able to explain the existing experimental observations. The model shows that the relationship between the relevant biochemical quantities can be viewed as strong linear amplification, where this effect is a consequence of fast non-specific degradation of pre-crRNA. This implicates that the unidentified nuclease, which is responsible for the non-specific degradation, is a major control element of CRISPR/Cas response. We furthermore pointed to the potential importance of regulation of CRISPR array transcription, which may be another important mechanism of CRISPR/Cas system induction. Elucidating how the system is induced under natural conditions remains a major question to be addressed by both experimental and theoretical research.

## Methods

### Overexpression of *cas* genes

Kinetic equations that describe generation, degradation and processing of CRISPR transcripts (see Figure 1) are given below:

(0.7) $$\frac{d\left[u\right]}{dt}=\phi -\lambda_{u}\left[u\right]-k\left[u\right]$$

(0.8) $$\frac{d\left[p\right]}{dt}=-\lambda_{p}\left[p\right]+k\left[u\right]\;$$

Notation used in the above equations is described in Results and Figure 1. In the steady state $d\left[u\right]/\mathrm{dt}=0$ and $d\left[p\right]/\mathrm{dt}=0$, so:

(0.9) $$0=\phi -\lambda_{u}\left[u\right]-k\left[u\right]\;$$

(0.10) $$0=-\lambda_{p}\left[p\right]+k\left[u\right]\;$$

Upon CasE overexpression, the new steady state becomes:

(0.11) $$0=\phi -\lambda_{u}\left[u\right]'-k'\left[u\right]'$$

(0.12) $$0=-\lambda_{p}\left[p\right]'+k'\left[u\right]'$$

In the above equations, note that upon CasE overexpression, CRISPR transcription rate *ϕ* and crRNA stability $\lambda_{p}$ do not change [11], while pre-crRNA processing rate increases to *k'*.

We next subtract Eq. (0.10) from Eq. (0.9) and subtract Eq. (0.12) from Eq. (0.11). We then again subtract these two expressions to obtain:

$$\lambda_{u}\left(\left[u\right]-\left[u\right]'\right)-\lambda_{p}\left(\left[p\right]'-\left[p\right]\right)=0$$

In the above expression, $\left[u\right]'-\left[u\right]$ is the change of pre-crRNA amount upon CasE overexpression, which we label as $\Delta \left[u\right]$. Similarly, we label the change of crRNA as $\Delta \left[p\right]=\left[p\right]'-\left[p\right]$. We therefore have:

(0.13) $$\Delta \left[p\right]=-\lambda_{u}/\lambda_{p}\Delta \left[u\right]$$

Furthermore, to calculate $\left[u\right]/\left[u\right]'$, we express [*u*] from Eq. (0.9) and [*u*]*'* from Eq. (0.11) to obtain:

(0.14) $$\frac{\left[u\right]}{\left[u\right]'}=\frac{\lambda_{u}+k'}{\lambda_{u}+k}∼10$$

Finally, we can solve for [*p*]*'* from Eqs. (0.11) and (0.12), and for [*p*] from Eqs. (0.9)and (0.10) to obtain:

(0.15) $$\frac{\Delta \left[p\right]}{\left[p\right]}=\frac{\lambda_{s}/k+1}{\lambda_{s}/k'+1}-1$$

### Joint increase of *cas* and CRISPR transcription

When transcription of both CRISPR and *cas* genes is increased, we assume that CRISPR transcription rate increases from *ϕ* to *ϕ'*, while pre-crRNA processing rate increases from *k* to *k'*. Then Eq. (0.11) and Eq. (0.12) become:

(0.16) $$0=\phi '-\lambda_{u}\left[u\right]'-k'\left[u\right]'$$

(0.17) $$0=-\lambda_{p}\left[p\right]'+k'\left[u\right]'$$

After expressing [*p*]*'* from Eqs. (0.16) and (0.17), and [*p*] from Eqs. (0.9) and (0.10) we obtain:

(0.18) $$\frac{\Delta \left[p\right]}{\left[p\right]}=\frac{\lambda_{s}/k+1}{\lambda_{s}/k'+1}\frac{\phi '}{\phi }-1$$

## Competing interests

The authors declare that they have no competing interests.

## Authors’ contributions

MD and KS conceived the work. MD and MD performed the analysis. MD wrote the paper, with the help of MD and KS. All authors read and approved the final manuscript.
